# Supplementary material for: Depletion of CD206+ M2-like macrophages induces fibro-adipogenic progenitors activation and muscle regeneration
Source: Nat Commun. 2022 Nov 21;13:7058. doi: 10.1038/s41467-022-34191-y (PMC9678897; doi:10.1038/s41467-022-34191-y)
Supplement: Supplementary file 1 — Supplementary Information [file 41467_2022_34191_MOESM1_ESM.pdf]

Depletion of CD206$^+$ M2-like macrophages induces fibro-adipogenic progenitors activation and muscle regeneration

Allah Nawaz[1,2,12*†], Muhammad Bilal[2], Shiho Fujisaka[2], Tomonobu Kado[2*], Muhammad Rahil Aslam[2], Saeed Ahmed[3], Yoshiko Igarashi[2], Keisuke Okabe[1,2], Yoshiyuki Watanabe[2], Takahide Kuwano[2], Koichi Tsuneyama[4], Ayumi Nishimura[2], Yasuhiro Nishida[2], Seiji Yamamoto[5], Masakiyo Sasahara[5], Johji Imura[6], Hisashi Mori[7], Martin M. Matzuk[8], Fujimi Kudo[9], Ichiro Manabe[9], Akiyoshi Uezumi[10], Takashi Nakagawa[1], Yumiko Oishi[11], Kazuyuki Tobe[2*]

Supplementary Figures 1-7

Supplementary Fig. 1

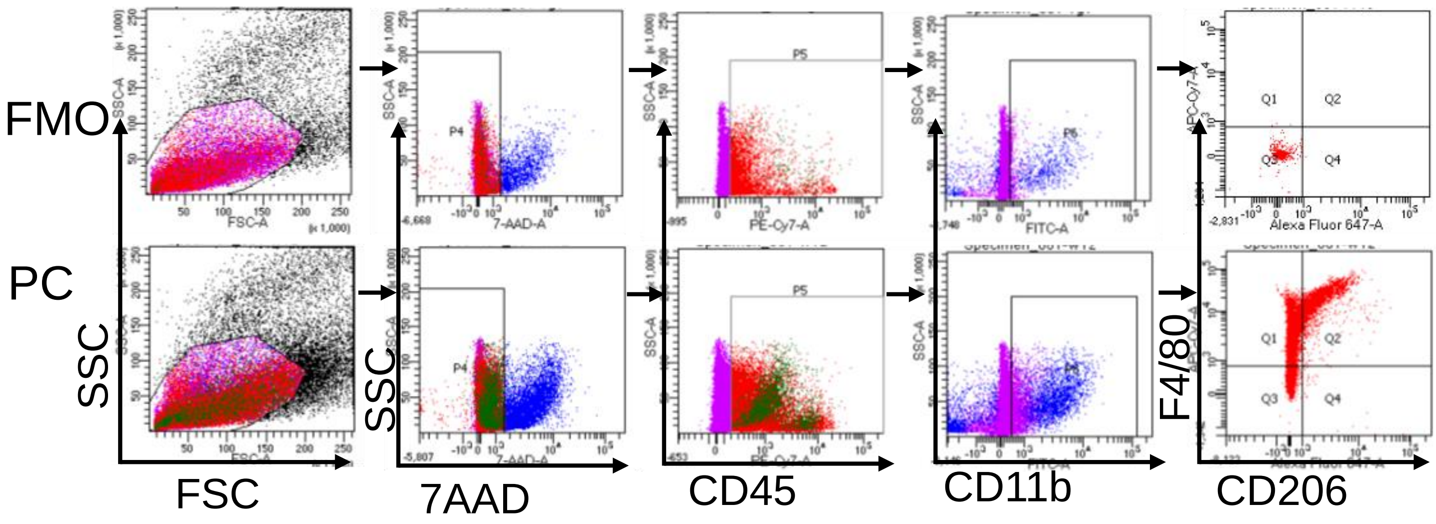

For identification of M2-like MΦ, the live cells were gated for $CD45^+$ cells, followed by positive selection of $CD11b^+$ and $F4/80^+CD206^+$ population. FMO; fluorescence minus one, PC; positive control

Supplementary Fig. 2

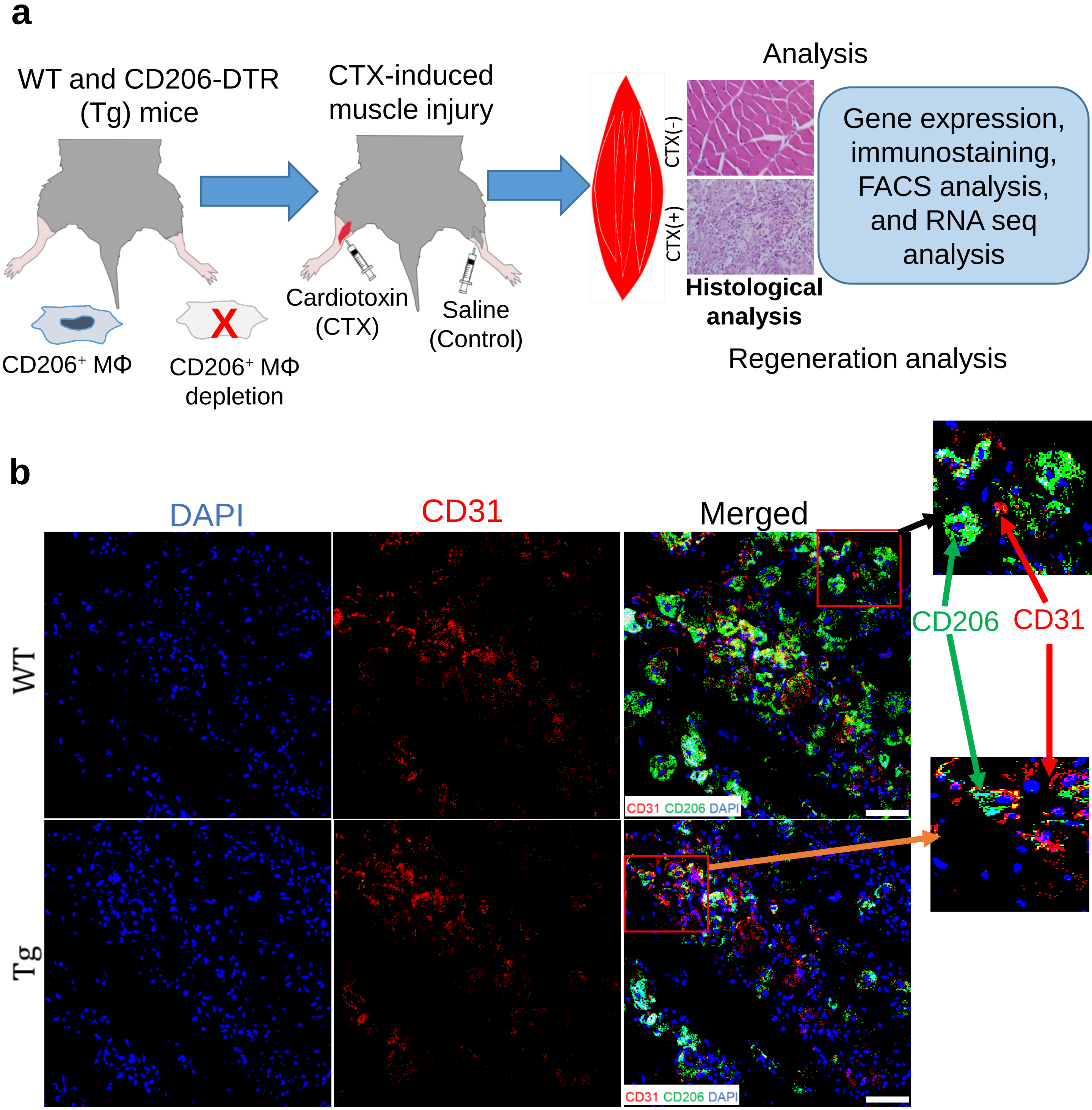

a. Schematic representation of CTX administration and tissue harvesting. TA and Gc muscles were harvested from Tg mice and control WT mice following acute injury by CTX-administration for analysis of muscle recovery.

b. Representative images of paraffin sections of muscle from Tg mice and WT control mice, harvested at 7 dpi, stained with anti-CD31 and anti-CD206 antibodies. (n=3-4 mice/group). Scale bar, 20 μm.

Supplementary Fig. 3

a

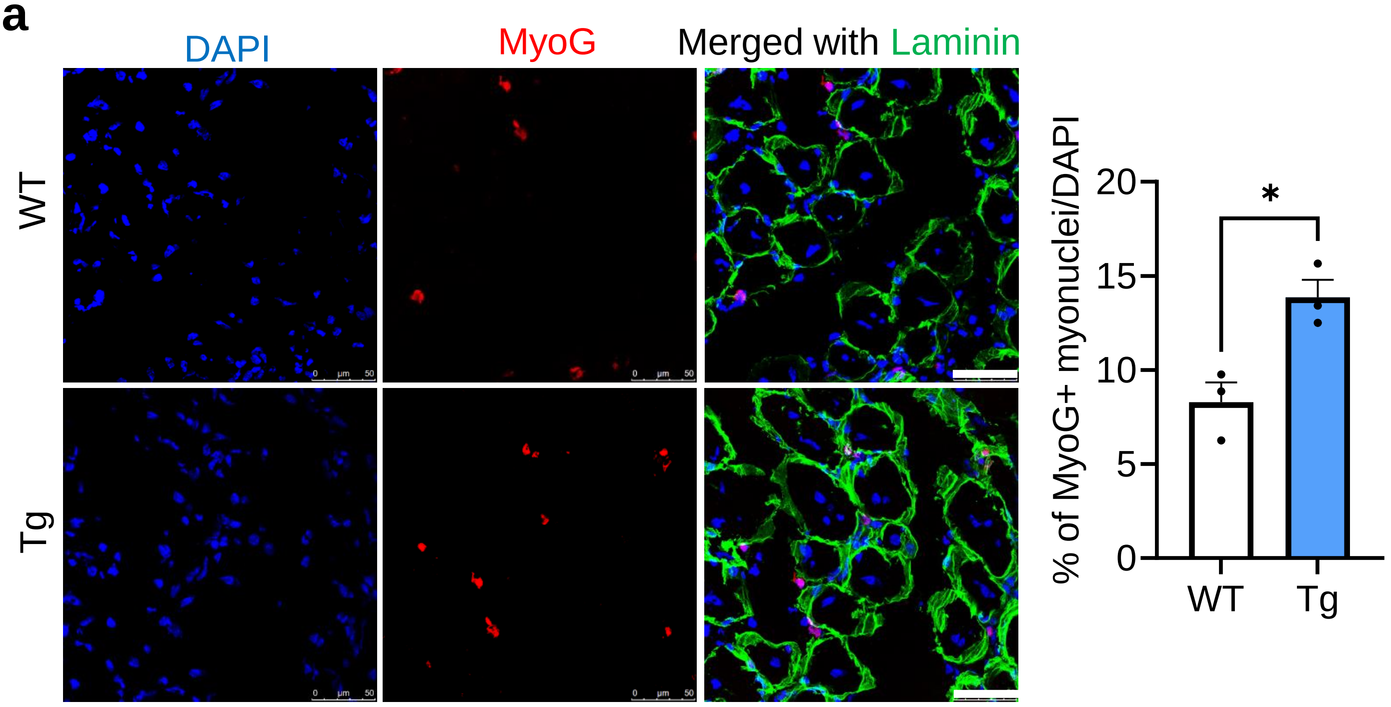

Representative images of frozen sections of muscle from WT and Tg mice harvested at 7 dpi stained with anti-laminin, and anti-MyoD antibodies (n=3 mice per group). Scale bar, 50 μm. Quantification is given in right panel. The data are shown as the means ± SEM. *$p < 0.05$ compared with littermate, two-tailed Student's t-test.

b

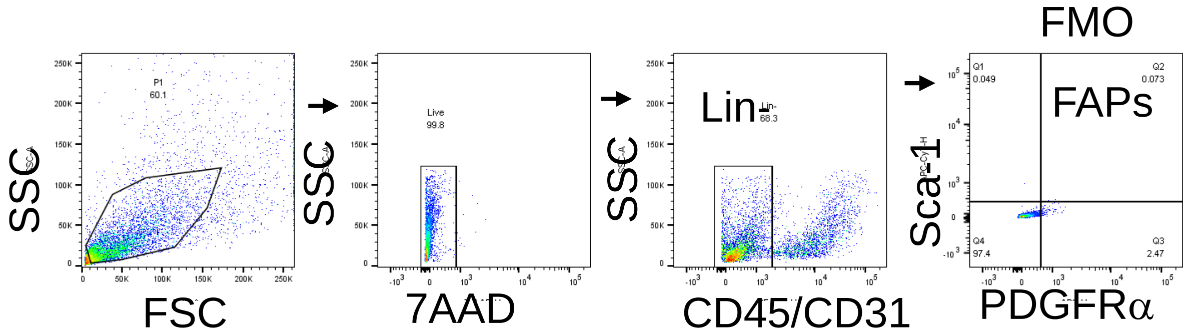

For FAPs, live cells were gated for negative selection of CD31 (endothelial) and CD45 (hematopoietic) cells followed by the positive selection of PDGFRα and Sca-1 double positive population.

c

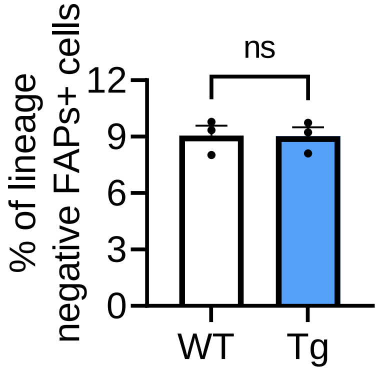

Quantification of lineage negative FAPs+ cells (n=3 mice per group). The data are shown as the means ± SEM. ns, p=0.972 compared with littermate, two-tailed Student's t-test.

Supplementary Fig. 4

a

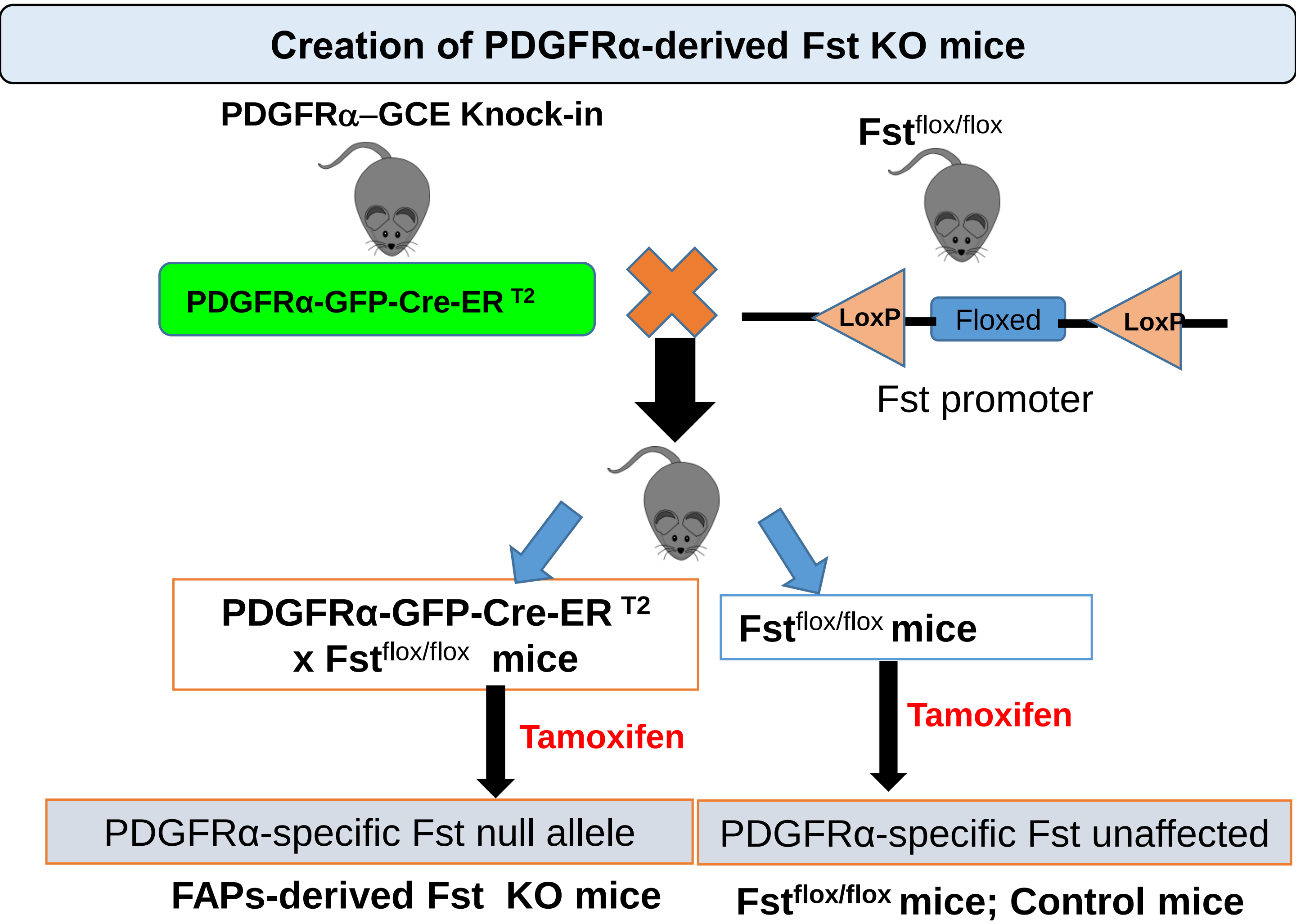

FAPs-derived Fst KO mice were created by crossing PDGFRα-GCE CreER^{T2} (PDGFRα–GCE) mice with Fst$^{f/f}$ mice.

b

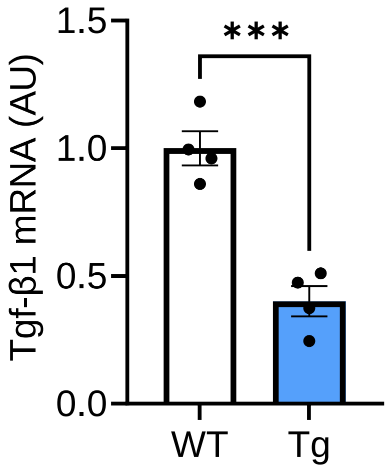

Relative mRNA expression of Tgf-β1 gene in FACS-isolated CD206^{+} M2-like macrophages of Tg mice compared to WT littermate control mice (n=4 mice per group). The data are shown as the means ± SEM. ***p <0.0001, compared with their littermates as determined using the two-tailed Student's t-test.

Supplementary Fig. 5

a

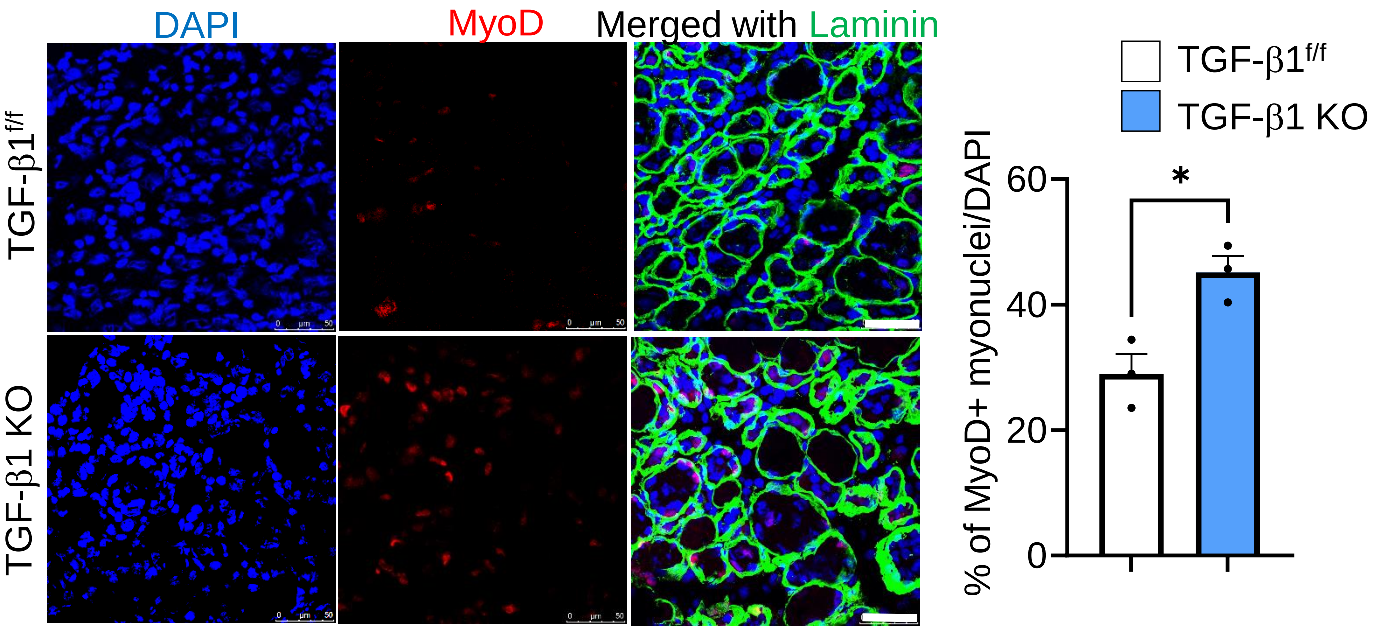

Representative images of frozen sections of muscle from TGF-β1 KO and TGF-β1$^{f/f}$ mice, harvested at 7 dpi stained with anti-laminin, and anti-MyoD antibodies (n=3 mice per group). Scale bar, 50 μm. Quantification is given in right panel (n=3 mice per group). The data are shown as the means ± SEM. *$p < 0.05$ compared with littermate as determined using the two-tailed Student's t-test.

b

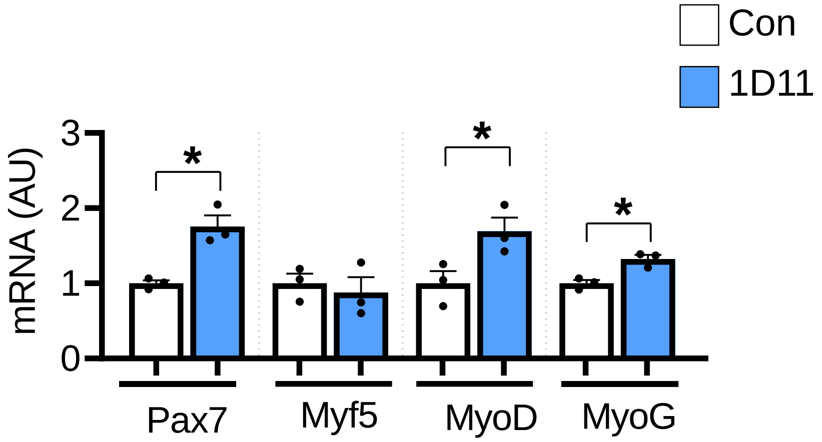

Relative mRNA expression levels of myogenesis-related marker genes in differentiated C2C12 myoblast treated with TGF-β 1,2,3 neutralizing (1D11) monoclonal antibody (n=3 wells per group). The data are shown as the means ± SEM. *$p < 0.05$ compared with control as determined using the two-tailed Student's t-test.

Supplementary Fig. 6

a

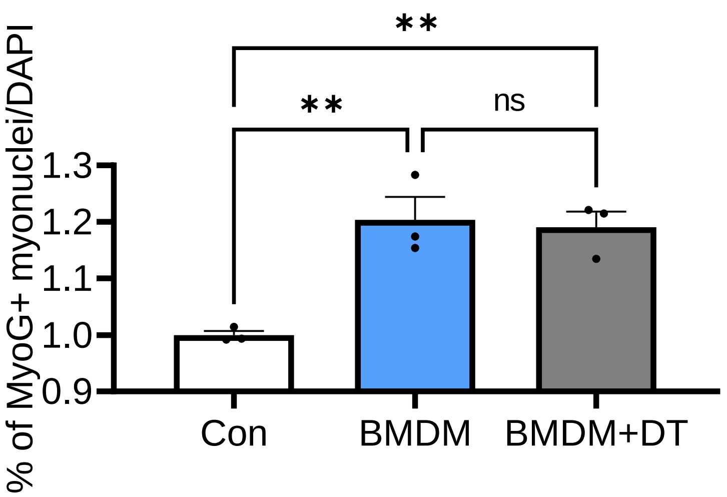

b

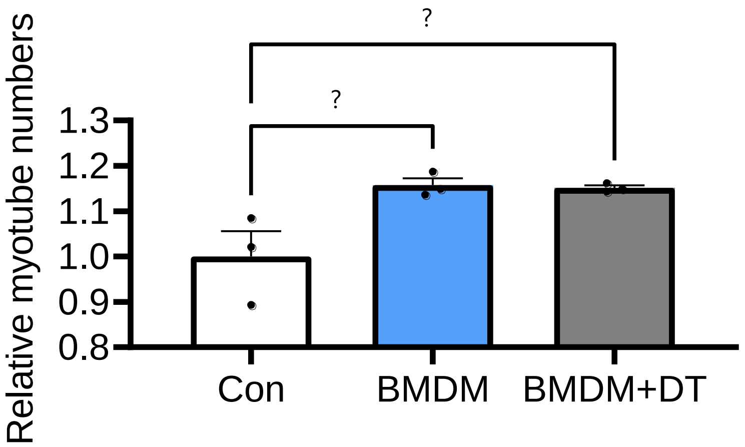

a, b. % of MyoG+ myonuclei and relative numbers in co-culture of BMDM and C2C12 myoblast (n=3 wells per treatment). The data are shown as the means ± SEM. *p < 0.05, **p < 0.01, and ns; non-significant as determined using the One-way Anova.

Supplementary Fig. 7

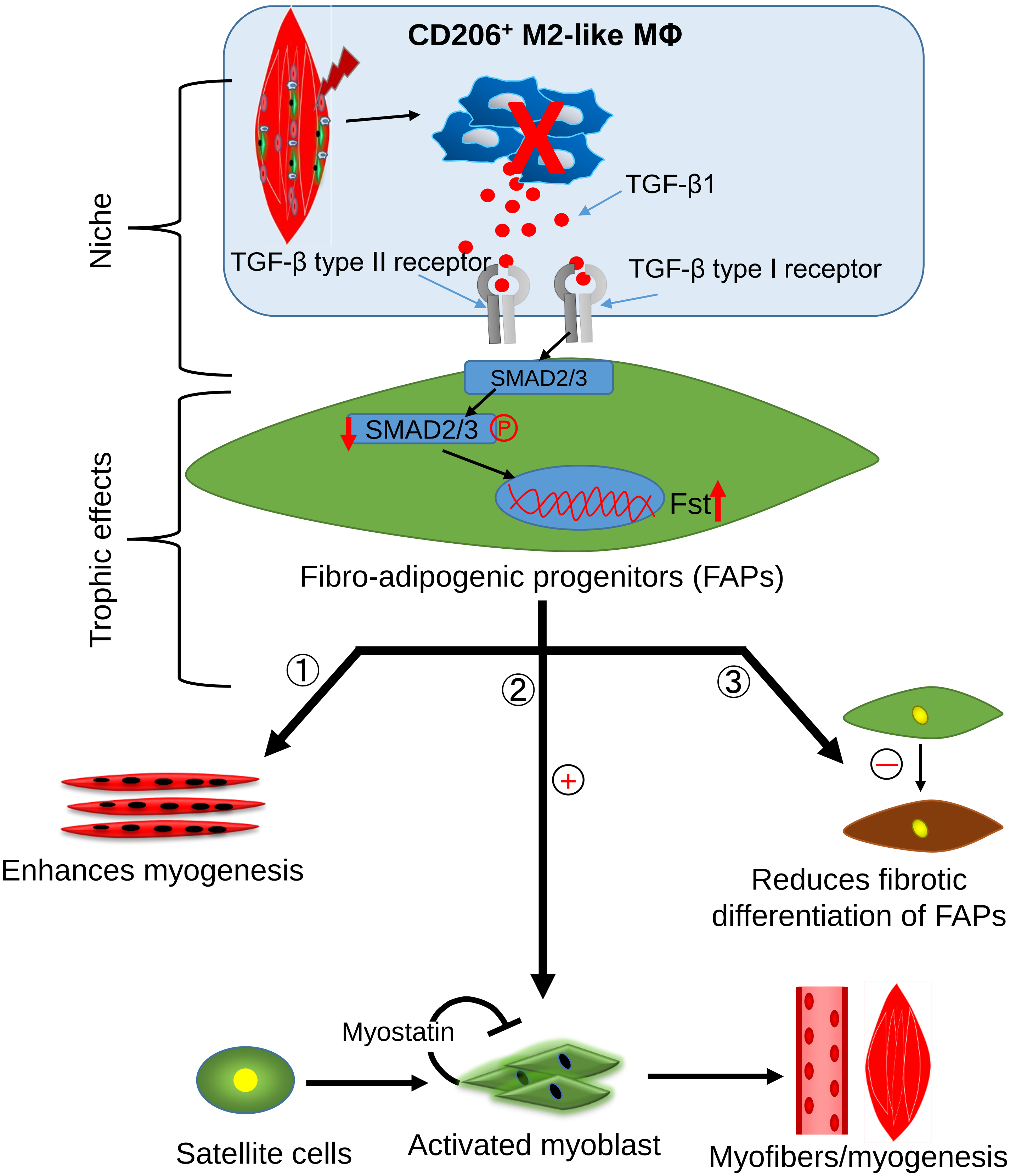

Supplementary Fig. 7; Schematic diagram showing crosstalk between CD206$^+$ M2-like MΦ and FAPs during the recovery process.

Inhibition of CD206$^+$ M2-like MΦ reduces TGF-β signaling and elevates FAPs-derived follistatin (Fst), thus promoting recovery of muscle after injury. Depletion of CD206$^+$ M2-like MΦ affects the early phase of the recovery process involving three possible pathways.

1. Depletion of CD206$^+$ M2-like MΦ or deletion of CD206$^+$ M2-like MΦ-specific TGF-β1 promote myogenesis.
2. CD206$^+$ M2-like MΦ-derived TGF-β1 inhibits the activation of FAPs, even during injury. Depletion of CD206$^+$ M2-like MΦ or deletion of the CD206$^+$ M2-like MΦ-specific TGF-β1 results in the activation of FAPs. Activated FAPs secrete Fst and Fstl3, which inhibit TGF-β/myostatin signaling in myoblast and myogenic precursors, thereby promoting myogenesis. FAP-derived Fst also promotes satellite cell differentiation into myoblast or myogenic progenitors, thereby mitigating the recovery process.
3. CD206$^+$ M2-like MΦ-derived TGF-β1 promotes fibrotic differentiation of FAPs into myofibroblasts. Either depletion of CD206$^+$ M2-like MΦ or deletion of CD206$^+$ M2-like MΦ-specific TGF-β1 attenuates fibrotic differentiation of FAPs, partly via activated FAPs, thereby secreting Fst and Fstl3, thus blocking TGF-β/myostatin signaling and promoting fibrosis-free healthy recovery from muscle injury.
