# Supplementary material for: Depletion of CD206+ M2-like macrophages induces fibro-adipogenic progenitors activation and muscle regeneration
Source: Nat Commun. 2022 Nov 21;13:7058. doi: 10.1038/s41467-022-34191-y (PMC9678897; doi:10.1038/s41467-022-34191-y)
Supplement: Supplementary file 3 — Reporting Summary [file 41467_2022_34191_MOESM3_ESM.pdf]

nature portfolio

Corresponding author(s): Kazuyuki Tobe, Allah Nawaz, and Tomonobu Kado

Last updated by author(s): Sep 22, 2022

Reporting Summary

Nature Portfolio wishes to improve the reproducibility of the work that we publish. This form provides structure for consistency and transparency in reporting. For further information on Nature Portfolio policies, see our Editorial Policies and the Editorial Policy Checklist.

Statistics

For all statistical analyses, confirm that the following items are present in the figure legend, table legend, main text, or Methods section.

n/a	Confirmed	
☐	☒	The exact sample size (*n*) for each experimental group/condition, given as a discrete number and unit of measurement
☐	☒	A statement on whether measurements were taken from distinct samples or whether the same sample was measured repeatedly
☐	☒	The statistical test(s) used AND whether they are one- or two-sided<br>*Only common tests should be described solely by name; describe more complex techniques in the Methods section.*
☒	☐	A description of all covariates tested
☒	☐	A description of any assumptions or corrections, such as tests of normality and adjustment for multiple comparisons
☐	☒	A full description of the statistical parameters including central tendency (e.g. means) or other basic estimates (e.g. regression coefficient) AND variation (e.g. standard deviation) or associated estimates of uncertainty (e.g. confidence intervals)
☐	☒	For null hypothesis testing, the test statistic (e.g. *F*, *t*, *r*) with confidence intervals, effect sizes, degrees of freedom and *P* value noted<br>*Give P values as exact values whenever suitable.*
☒	☐	For Bayesian analysis, information on the choice of priors and Markov chain Monte Carlo settings
☒	☐	For hierarchical and complex designs, identification of the appropriate level for tests and full reporting of outcomes
☒	☐	Estimates of effect sizes (e.g. Cohen's *d*, Pearson's *r*), indicating how they were calculated

Our web collection on statistics for biologists contains articles on many of the points above.

Software and code

Policy information about availability of computer code

Data collection	RNAseq analysis; RNA-seq libraries were prepared using an RNA-Seq library preparation kit for Illumina (New England Biolabs), The reads were aligned to the mm9 mouse genome using STAR. Aligned read files were analyzed using HOMER. Differentially expressed genes were analyzed using DESeq2. K-means clustering was performed using Cluster 3.0. Gene set analysis was performed using Metascape. R software and RStudio was used to analyze and prepare heatmap of RNA seq data.<br>Microsoft Office Excel 2013.
Data analysis	ImageJ Software (11.53a), National Institute of Health, USA.<br>FlowJo version 8 (FlowJo https://www.flowjo.com/).<br>GraphPad Prism9 (GraphPad software for Windows).<br>R Project for Statistical Computing (https://www.r-project.org) R software and RStudio (version 1.2.5033).

For manuscripts utilizing custom algorithms or software that are central to the research but not yet described in published literature, software must be made available to editors and reviewers. We strongly encourage code deposition in a community repository (e.g. GitHub). See the Nature Portfolio guidelines for submitting code & software for further information.

Data

Policy information about availability of data

All manuscripts must include a data availability statement. This statement should provide the following information, where applicable:

- Accession codes, unique identifiers, or web links for publicly available datasets
- A description of any restrictions on data availability
- For clinical datasets or third party data, please ensure that the statement adheres to our policy

RNA-seq of WT and CD206DTR (Tg) mice after CTX injury (GEO; GSE173555) and RNA-seq of isolated FAPs of WT and CD206DTR mice after CTX injury (GEO; GSE173555) were deposited.

Human research participants

Policy information about studies involving human research participants and Sex and Gender in Research.

Reporting on sex and gender	N/A
Population characteristics	N/A
Recruitment	N/A
Ethics oversight	N/A

Note that full information on the approval of the study protocol must also be provided in the manuscript.

Field-specific reporting

Please select the one below that is the best fit for your research. If you are not sure, read the appropriate sections before making your selection.

☒ Life sciences ☐ Behavioural & social sciences ☐ Ecological, evolutionary & environmental sciences

For a reference copy of the document with all sections, see nature.com/documents/nr-reporting-summary-flat.pdf

Life sciences study design

All studies must disclose on these points even when the disclosure is negative.

Sample size	Sample size of each experiment is given in the figure legends. Sample size was determined by preliminary experiments. The sample size for our animal experiments in this study was based on our experience with experimental models, anticipated variables and previous literature. For experiments involving quantification of temporal recovery analysis at D14 (n=2), 0, 4, and 7 (n=4). For experiments of For T-PCR n=4 (WT), n=5 (Tg), flow cytometry n=3, histology n=3, RNA-seq n=2, and n=analysis of regeneration at 7 dpi, n=3 was chosen and sample size was determined by the number of centrally nucleated myofibers. n=46 was chosen for RT-PCR analysis at 7dpi, n=3 for flow cytometry analysis and gene expression of FACS-isolated samples, n=3-4 for all histological sections, n=2 for total RNA seq, Ex vivo n=3 and in vitro n=4.
Data exclusions	Data were not excluded from analysis.
Replication	All the biological experiments were repeated, at least, twice and reproduced. RNA seq were performed once but 2 independent samples were analyzed and further validated by alternative approaches, such as RT-PCR. Technical replicated were used for in vitro and ex vivo. All replication attempts were successful. All measure were replicated at least 3 times unless stated otherwise in the figure legends.
Randomization	All cells were randomly assigned to experimental group. All animals were randomly selected for each group by age and body weight. Random images were taken focusing on injured and regenerated area and this was same for all specimens. We excluded uninjured area for quantification.
Blinding	RNA sequencing and library constructions were performed by collaborators who were blinded to experimental groups. RNA sequencing alignment and analyses were performed by the authors who were blinded to mouse genotype. Rest of the experiments in our work were based on comparative analysis and investigators were not needed to distinguish between control and test group.

Reporting for specific materials, systems and methods

We require information from authors about some types of materials, experimental systems and methods used in many studies. Here, indicate whether each material, system or method listed is relevant to your study. If you are not sure if a list item applies to your research, read the appropriate section before selecting a response.

Materials & experimental systems

n/a	Involved in the study
	☒ Antibodies
	☒ Eukaryotic cell lines
☒	☐ Palaeontology and archaeology
	☒ Animals and other organisms
☒	☐ Clinical data
☒	☐ Dual use research of concern

Methods

n/a	Involved in the study
☒	☐ ChIP-seq
	☒ Flow cytometry
☒	☐ MRI-based neuroimaging

Antibodies

Antibodies used	The following antibodies were used in this study:<br>Anti-TGF-β1 (Cat# Sc-146, dilution 1:100) antibody was obtained from Santa Cruz Biotechnology (Dallas, Texas);<br>rabbit monoclonal anti-MyoD1 (ab133627, clone# EPR6653-131, dilution 1:200), and rabbit monoclonal anti-Myogenin (ab124800, clone# EPR4789, dilution 1:200) antibodies were purchased from Abcam,<br>rat monoclonal anti-Laminin alpha2 (Sc-59854, clone# 4H8-2, dilution 1:200) antibody was purchased from Santa Cruz,<br>anti- p27kip1 (Cat# 3698, dilution 1:100) antibody was purchased from Cell Signaling Technology.<br>Myosin heavy chain (embryonic) (MyH3) antibody (F1.652, 3 ug/ml) antibody was purchased from Developmental Studies Hybridoma Bank (DSHB).<br>Monoclonal mouse anti-TGF-β1, 2, and 3 (1D11, 0.5 ug/mL) (Cat#MAB1835, Lot# CCI1216031) antibody was purchased from R&D Systems.<br>APC/Cy7 anti-mouse Ly-6A/E (Sca-1) (Cat# 108125, Clone: D7, dilution 1:400),<br>APC anti-mouse CD140a (Cat# 135907, Clone: APA5, dilution 1:100),<br>APC-Cy7 anti-mouse F4/80 (Cat# 123118, Clone: BM8, dilution 1:100), and<br>APC anti-mouse CD206 (MMR) (Cat# 141707, MCA2235, Clone: MR5D3, dilution 1:100) antibodies were purchased from Biolegend.<br>PE-CY7 anti-mouse CD31 (Cat#25-0311-82, Clone: 390, dilution 1:400), PE-CY7 anti-mouse CD45 (Cat#25-0451-82, Clone: 30-F11, dilution 1:400), and FITC anti-mouse CD11b (Cat#11-0112-81, Clone: M1/70, dilution 1:100) antibodies were purchased from eBioscience.
Validation	All antibodies used in this study were commercially developed and used in previous studies. Most antibodies were also used and validated in our previous work. Information regarding validation and application can be found on manufacturer's website.<br>-Anti-TGF-β1 (Cat# Sc-146, 1:100),<br>-Monoclonal mouse anti-TGF-β1, 2, and 3 (1D11) (Cat#MAB1835), and<br>-Anti- p27kip1 (Cat# 3698, 1:100) were validated in our previous work (Nawaz et al., 2017).<br>https://datasheets.scbt.com/sc-146.pdf,<br>https://www.rndsystems.com/products/tgf-beta1-2-3-antibody-1d11_mab1835#product-datasheets, and https://www.cellsignal.com/product/productDetail.jsp?productId=3698.<br>-Anti-MyoD1 (ab133627, 1:200), https://www.abcam.co.jp/myod1-antibody-epr6653-131-ab133627.html,<br>-Anti-Myogenin (ab124800, 1:200), https://www.abcam.co.jp/myogenin-antibody-epr4789-ab124800.html,<br>-Anti-MyH3 (F1.652, 3 ug/ml), https://dshb.biology.uiowa.edu/F1-652, and<br>-Anti-Laminin alpha2 (sc-59854, 1:200), https://www.scbt.com/ja/p/laminin-alpha-2-antibody-4h8-2, antibodies were validated in our previous work (Uezumi, A et al., 2010 and 2021).<br>-APC/Cy7 anti-mouse Ly-6A/E (Sca-1) (Cat# 108125, Clone:D7), https://www.biolegend.com/en-us/products/apc-cyanine7-anti-mouse-ly-6a-e-sca-1-antibody-6752,<br>-APC anti-mouse CD140a (Cat# 135907, Clone: APA5), https://www.biolegend.com/en-us/products/apc-anti-mouse-cd140a-antibody-6439,<br>-APC-Cy7 anti-mouse F4/80 (Cat# 123118, Clone: A3-1), https://www.biolegend.com/en-us/products/apc-cyanine7-anti-mouse-f4-80-antibody-4072,<br>-APC anti-mouse CD206 (MMR) (Cat# 141707, MCA2235, Clone: MR5D3), https://www.biolegend.com/en-us/products/apc-anti-mouse-cd206-mmr-antibody-7425,<br>-PE-CY7 anti-mouse CD31 (Cat# 25-0311-82, Clone: 390), https://www.thermofisher.com/antibody/product/CD31-PECAM-1-Antibody-clone-390-Monoclonal/25-0311-82,<br>-PE-CY7 anti-mouse CD45 (Cat# 25-0451-82), https://www.thermofisher.com/antibody/product/CD45-Antibody-clone-30-F11-Monoclonal/25-0451-82,<br>-FITC anti-mouse CD11b (Cat# 11-0112-81, Clone: M1/70), https://www.thermofisher.com/antibody/product/CD11b-Antibody-clone-M1-70-Monoclonal/11-0112-81, antibodies were also used in our previous work (Nawaz A et al., 2017; Igarashi Y and Nawaz A et al., 2018).

Eukaryotic cell lines

Policy information about cell lines and Sex and Gender in Research

Cell line source(s)	Primary myoblast from cardiotoxin-induced muscle of C57BL/6J mice, and C2C12 mouse myoblasts cell line was obtained from American Type Culture Collection (Manassas, Virginia).
Authentication	C2C12 cell line was authenticated in Dr Tobe lab (Nishida Y, and Nawaz A, et al., 2020).
Mycoplasma contamination	Mycoplasma free cells were provided by the supplier. Cell line was not tested for mycoplasma contamination but no indication of contamination was observed.

March 2021

Commonly misidentified lines (See ICLAC register)	No commonly misidentified cell lines were used.

Animals and other research organisms

Policy information about studies involving animals; ARRIVE guidelines recommended for reporting animal research, and Sex and Gender in Research

Laboratory animals	Male C57BL/6 mice (age 12 weeks), CD206-DTR (Tg) and their littermate control mice of age 12 weeks, CD206-CreERT2 x TGF-b1 KO and their littermate control TGF-b1 flox/flox mice of age 14 weeks, FAPs-specific Fst KO mice and their littermate control Follistatin flox/flox mice of age 14 weeks were used in respective experiments. All animals were housed under a 12-h light/12-h dark cycle, room temperature 22°C, and humidity of 45% ± 5 and were allowed ad libitum access to water and standard chow diet (Nosan Corporation, Yokohama, Japan), and all animals were transferred to clean cages once weekly.
Wild animals	No wild animals were used in this study.
Reporting on sex	We used only male animals in this study.
Field-collected samples	No field-collected samples were used in this study.
Ethics oversight	All animal care policies and protocols for the experiments were approved by the Animal Experiment Committee at the University of Toyama, Toyama, Japan (Authorization No. A2019MED-7, and A2022MED-21).

Note that full information on the approval of the study protocol must also be provided in the manuscript.

Flow Cytometry

Plots

Confirm that:

☒ The axis labels state the marker and fluorochrome used (e.g. CD4-FITC).

☒ The axis scales are clearly visible. Include numbers along axes only for bottom left plot of group (a 'group' is an analysis of identical markers).

☒ All plots are contour plots with outliers or pseudocolor plots.

☒ A numerical value for number of cells or percentage (with statistics) is provided.

Methodology

Sample preparation	Isolation and separation of single cells and subsequent flow cytometry were performed as previously described (Joe et al., 2010 and Uezumi et al., 2010) with minor modifications. Flow cytometry for the detection of FAPs was performed in a manner similar to a previously reported method. First, the negative selection of CD31+ (endothelial) and CD45+ (hematopoietic) cells was performed, followed by positive selection of Sca-1+/PDGFRa+ cells. The purified cells were subjected to RT-PCR, total RNA-seq analysis and these purified cells were also proceeded for ex vivo co-culture experiments.
Instrument	This experiment was performed using a FACSDiva Version 6.1.2 automated cell analyzer (Becton Dickinson FACSCanto II) and an automatic cell sorting analyzer (Becton Dickinson FACSAria SORP).
Software	The data were analyzed using the FlowJo software.
Cell population abundance	Viable cells were defined using FSc/SSc characteristics and gated for abundance of FAPs populations and sorted using 4-way purity without loss of target cells.
Gating strategy	Viable cells were defined using FSc/SSc, and gated for live cells in all condition. For isolation of abundance of FAPs populations, live cells were gated for negative selection of CD31 and CD45 and then for positive selection of Sca-1+PDGFRa+ populations. For isolation of macrophages, live cells were gated for positive selection of CD45, then CD11b population. CD11b+ population was gated for positive selection of F4/80 and CD206+ cells as M2-like macrophages.

☒ Tick this box to confirm that a figure exemplifying the gating strategy is provided in the Supplementary Information.
